# MPN-RRT*: A New Method in 3D Urban Path Planning for UAV Integrating Deep Learning and Sampling Optimization

**DOI:** 10.3390/s25134142

**Published:** 2025-07-02

**Authors:** Yue Zheng, Ang Li, Zihan Chen, Yapeng Wang, Xu Yang, Sio-Kei Im

**Affiliations:** Faculty of Applied Sciences, Macao Polytechnic University, Macao 999078, China; p2411653@mpu.edu.mo (Y.Z.); p2415552@mpu.edu.mo (A.L.); p2213111@mpu.edu.mo (Z.C.); xuyang@mpu.edu.mo (X.Y.); marcusim@mpu.edu.mo (S.-K.I.)

**Keywords:** deep learning, path planning, UAV, MPNet, RRT*, MPN-RRT*

## Abstract

The increasing deployment of unmanned aerial vehicles (UAVs) in complex urban environments necessitates efficient and reliable path planning algorithms. While traditional sampling-based methods such as Rapidly exploring Random Tree Star (RRT*) are widely adopted, their computational inefficiency and suboptimal path quality in intricate 3D spaces remain significant challenges. This study proposes a novel framework (MPN-RRT*) that integrates Motion Planning Networks (MPNet) with RRT* to enhance UAV navigation in 3D urban maps. A key innovation lies in reducing computational complexity through dimensionality reduction, where 3D urban terrains are sliced into 2D maze representations while preserving critical obstacle information. Transfer learning is applied to adapt a pre-trained MPNet model to the simplified maps, enabling intelligent sampling that guides RRT* toward promising regions and reduces redundant exploration. Extensive MATLAB simulations validate the framework’s efficacy across two distinct 3D environments: a sparse 200 × 200 × 200 map and a dense 800 × 800 × 200 map with no-fly zones. Compared to conventional RRT*, the MPN-RRT* achieves a 47.8% reduction in planning time (from 89.58 s to 46.77 s) and a 19.8% shorter path length (from 476.23 m to 381.76 m) in simpler environments, alongside smoother trajectories quantified by a 91.2% reduction in average acceleration (from 14.67 m/s² to 1.29 m/s²). In complex scenarios, the hybrid method maintains superior performance, reducing flight time by 14.2% and path length by 13.9% compared to RRT*. These results demonstrate that the integration of deep learning with sampling-based planning significantly enhances computational efficiency, path optimality, and smoothness, addressing critical limitations in UAV navigation for urban applications. The study underscores the potential of data-driven approaches to augment classical algorithms, providing a scalable solution for real-time autonomous systems operating in high-dimensional dynamic environments.

## 1. Introduction

In the rapidly advancing era of automation and robotics, path planning has emerged as a cornerstone technology for navigating complex environments. Autonomous vehicles, drones, and industrial robots rely on efficient path planning algorithms to traverse urban roadways, aerial spaces, and manufacturing facilities while optimizing performance metrics such as travel time and energy consumption. As environments grow in complexity and dynamism, particularly in three-dimensional urban settings, traditional path planning algorithms face significant challenges in generating efficient and reliable trajectories. This study explores the integration of Motion Planning Networks (MPNet), a deep learning-based approach, to enhance the performance of sample-based path planning algorithms such as Rapidly exploring Random Trees Star (RRT*) in 3D urban environments. environments.

### 1.1. Objective

The primary objective of this research is to optimize sample-based path planning algorithms in 3D urban environments by leveraging MPNet. By combining MPNet’s deep learning capabilities with the traditional RRT* algorithm, this study aims to improve path planning efficiency and accuracy. Through simulations in MATLAB, this research investigates how dimensionality reduction and data augmentation techniques applied to 3D maps can enhance model training and path planning performance.

### 1.2. Background

Path planning is fundamental to robotics and autonomous systems, requiring algorithms to navigate from a start point to a destination while avoiding obstacles. Traditional algorithms like RRT and RRT* have demonstrated effectiveness in high-dimensional spaces but struggle with computational demands in dynamic and complex environments. RRT explores spaces through random sampling, while RRT* introduces path optimization, iteratively refining the path to approach optimality. However, these algorithms often require significant computational resources, particularly in 3D urban settings.

MPNet represents a significant advancement by integrating deep learning with traditional path planning. Trained on diverse environments, MPNet can predict feasible paths efficiently, even in previously unseen contexts. This capability is particularly valuable in dynamic environments where traditional algorithms may struggle with computational complexity. MPNet’s ability to generalize across different environments makes it a promising solution for real-time navigation in complex and changing settings.

### 1.3. Methodology Overview and Significance

This study employs a methodological approach that combines dimensionality reduction, deep learning, and traditional path planning. By transforming complex 3D urban maps into simplified 2D maze maps, this research streamlines the training process for MPNet. This dimensional reduction technique preserves essential navigational features while simplifying the computational complexity. The MPNet model is then trained using these 2D representations to learn optimal pathfinding strategies. The trained MPNet is integrated with RRT* to enhance its sampling process, guiding the algorithm towards more promising areas and reducing ineffective sampling.

Simulation results demonstrate a marked improvement in path planning performance when using MPN-RRT*. Key metrics such as path length, planning time, and path smoothness show significant enhancements. These findings underscore the effectiveness of combining deep learning with traditional path planning algorithms, highlighting the potential for more efficient and reliable navigation in complex urban environments.

The integration of MPNet with RRT* represents a significant advancement in path planning for 3D urban environments. This innovative approach not only enhances the efficiency and reliability of path planning but also opens new avenues for autonomous navigation in complex and dynamic settings. Future research will focus on expanding the applicability of this framework to diverse environments and further refining the generalization capabilities of MPNet.

### 1.4. Summary

Our work makes the following key contributions to the field of UAV path planning in complex 3D urban environments:

1. We propose a novel path planning framework named MPN-RRT*, which is the first to combine Motion Planning Networks (MPNet) with RRT*. This integration leverages the deep learning capabilities of MPNet to enhance the sampling efficiency and path quality of the traditional RRT* algorithm.
2. We introduce an innovative dimensionality reduction technique that transforms complex 3D urban terrains into 2D maze representations. This method preserves critical obstacle information while significantly reducing computational complexity, making it more efficient for path planning tasks.
3. We apply transfer learning to adapt a pre-trained MPNet model to the simplified 2D maps. This allows the model to generate more intelligent and targeted sampling points, guiding the RRT* algorithm towards promising regions and reducing redundant exploration.
4. We develop a bidirectional neural planning approach within the MPN-RRT* framework. This approach generates paths from both the start to the goal and the goal to the start, improving the efficiency and effectiveness of the path search process.
5. Through extensive MATLAB simulations across two distinct 3D environments (a sparse 200 × 200 × 200 map and a dense 800 × 800 × 200 map with no-fly zones), we demonstrate the superior performance of MPN-RRT* compared to conventional RRT*. Our results show significant improvements in planning time, path length, and path smoothness, with specific metrics including a 47.8% reduction in planning time and a 19.8% shorter path length in simpler environments, as well as a 14.2% reduction in flight time and a 13.9% shorter path length in more complex scenarios.

These contributions together promote the development of UAV path planning technology in complex three-dimensional cities, demonstrating the great potential of the combination of deep learning and sampling algorithms.

## 2. Literature Review

Path planning is a critical functionality in automation and intelligent systems, essential for the safe and efficient navigation of robots, UAVs, and autonomous vehicles in complex environments. This section reviews key literature in path planning, focusing on sampling-based algorithms like RRT and RRT*, which excel in high-dimensional spaces due to their exploration mechanisms and efficiency. These algorithms form the foundation for addressing complex planning challenges.

RRT and RRT* are analyzed for their principles, improvements, and limitations. RRT rapidly explores spaces through random sampling, while RRT* enhances path quality by optimizing the search tree. However, their sampling processes can be inefficient in complex environments. MPNet, a deep learning technique, optimizes the sampling process by learning from complex path planning tasks, reducing ineffective sampling and accelerating path optimization. This integration of MPNet with RRT algorithms significantly improves path planning efficiency and quality, demonstrating the potential of deep learning in traditional path planning.

Overall, this study combines the exploration capabilities of RRT* with the intelligent sampling guidance of MPNet, advancing path planning methods for complex environments.

### 2.1. Fundamentals and Evolution of Path Planning

#### 2.1.1. Definition and Importance of Path Planning

Path planning is a core technology in robotics and autonomous systems, aiming to find a safe and efficient path for a mobile agent (such as a UAV, autonomous vehicle, or robot) from a starting point to a destination. As technology advances, path planning in complex environments, such as three-dimensional urban settings, dynamic obstacles, and resource-constrained scenarios, has become increasingly important. Path planning not only avoids collisions but also optimizes performance metrics such as path length, time, and energy consumption. Its applications span UAV navigation, autonomous driving, industrial robotics, disaster response, and space exploration.

#### 2.1.2. Sampling-Based Path Planning Algorithms

Sampling-based path planning algorithms [1] have proven effective for navigating complex, high-dimensional environments where traditional grid-based and graph-based methods face computational and scalability challenges. These algorithms randomly sample the state space and connect samples to form obstacle-free paths, offering computational efficiency and suitability for real-time applications with dynamic changes. They are particularly advantageous when precise geometric modeling is difficult or unnecessary.

RRT (Rapidly exploring Random Tree) efficiently explores high-dimensional spaces by growing a tree from the initial position towards random points. It is simple and quick but may produce non-optimal paths. RRT* (RRT Star), an enhanced version of RRT, incorporates a rewiring process to optimize the path as the tree expands. It aims to find the shortest route by evaluating neighbors of new nodes, though this increases computational complexity.

Sampling-based algorithms excel in handling complex environments and both static and dynamic obstacles. They are widely used in robotics, autonomous vehicles, game development, and virtual reality for their ability to provide quick, feasible solutions. However, the randomness in sampling can lead to solution variability, and the algorithms’ efficiency is sensitive to sampling strategies and density. RRT*, while optimizing paths, may impact real-time applicability due to higher computational demands.

These algorithms are indispensable in autonomous navigation across various fields. S. Karaman and E. Frazzoli [2] note progress in sampling-based optimal path planning but highlight challenges like high-dimensional state spaces and real-time performance. Klemm S, Hermann A et al. [3] propose RRT-Connect for faster planning while maintaining optimality. Future research directions include improving algorithm efficiency, handling dynamic environments, and integrating machine learning techniques [4]. J. Chen and J. Yu [5] enhance traditional RRT for better security and efficiency, while Zammit, Christian, and Erik-Jan Van Kampen [6] compare A* and RRT algorithms in 3D settings, emphasizing efficient routing needs. D. Lee, H. Song, and D. H. Shim [7] introduce spline-RRT* for fixed-wing UAVs, focusing on optimization and efficiency.

In summary, sampling-based path planning algorithms are crucial for autonomous navigation, but their application requires balancing solution quality, computational efficiency, and real-time performance.

#### 2.1.3. Motion Planning Networks (MPNet)

MPNet [8,9,10] has emerged as a groundbreaking approach that integrates deep learning with traditional path planning to address challenges in complex environment navigation. By leveraging neural networks’ ability to learn from extensive datasets, MPNet efficiently predicts collision-free trajectories, diverging from conventional sampling-based or optimization-based algorithms. This innovation introduces a new paradigm for rapid and efficient path planning, particularly in high-dimensional and dynamic environments.

MPNet was developed to overcome the limitations of traditional path planning algorithms, such as high computational costs and inefficiencies in dynamic environments. Its core structure consists of an encoder network that captures environmental context and a planning network that predicts feasible paths based on the current state and goal. The encoder simplifies the complexity faced by the planning network by effectively representing obstacles and free spaces.

A significant contribution of MPNet is its generalization capability across diverse environments. Unlike traditional algorithms that require reconfiguration for each new setting, MPNet can be applied to unseen environments once trained, making it highly adaptable. This feature is particularly valuable in robotics and autonomous vehicles, where rapid environmental adaptation is essential. MPNet has been successfully implemented in various fields, including autonomous driving, drone navigation, and robotic arm manipulation. It aids vehicles in navigating complex urban environments, enables efficient path planning for drones in cluttered areas, and assists robotic arms in manipulating objects in densely packed spaces.

Despite its benefits, MPNet faces challenges related to the diversity and quality of training data, which critically impact path planning effectiveness. Additionally, integrating MPNet into real-time systems is challenging due to the computational demands of deep neural networks.

Future research will focus on enhancing MPNet’s efficiency, scalability, and generalization capabilities, as well as integrating it with reinforcement learning to improve adaptability and performance in dynamic settings. Recent advancements highlight MPNet’s potential in optimizing sample-based path planning algorithms for 3D urban maps, building on work by Spyridon [11], Jianqing [5], and Marco Dorigo [12], which underscore neural planners’ efficiency in high-dimensional spaces and their ability to generalize. These studies indicate a promising direction for overcoming current motion planning limitations in complex urban environments.

## 3. Methodology

This section proposes a hybrid MPN-RRT* algorithm combining Motion Planning Networks (MPNet) and Rapidly exploring Random Tree Star (RRT*) for UAV path planning in complex 3D cities. Through the combination of deep learning and a sampling algorithm, the algorithm significantly improves the efficiency of path planning, the optimality, and performance of the path.

Traditional RRT and RRT* algorithms typically employ uniform or biased random sampling strategies to explore potential paths within the configuration space. While these random sampling methods are simple, they are often inefficient, particularly in complex or high-dimensional environments. Random sampling often requires a large number of iterations to identify satisfactory paths, which is especially detrimental in real-time or resource-constrained application scenarios. In contrast, MPNet, a deep learning-based path planning optimization technique, can significantly enhance sampling efficiency. By learning the distribution of obstacles and feasible path characteristics within the configuration space, MPNet can direct the sampling process towards more promising areas, thereby reducing unnecessary sampling and accelerating the path search process. When integrating MPNet into the sampling component of RRT* algorithm, it can expect faster simulation times, shorter path lengths, and improved path smoothness. This improvement stems from MPNet’s ability to bypass the randomness inherent in traditional sampling methods, strategically placing sampling points in regions critical for path discovery. This optimization not only enhances the efficiency and effectiveness of path planning but also highlights the potential of deep learning to augment traditional path planning algorithms.

### 3.1. MPN-RRT* Algorithm

**Figure 1:**
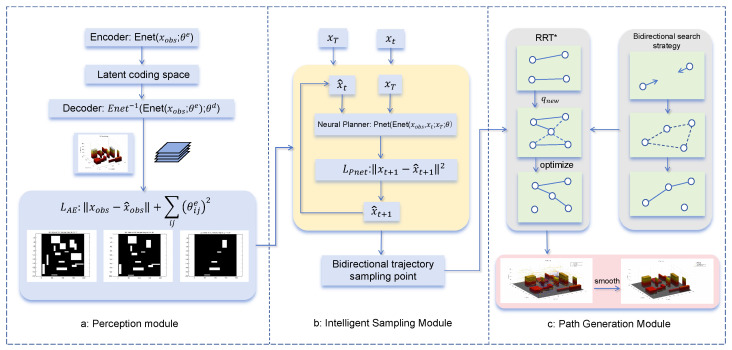
(**a**) The 3D map is converted to 2D for dimensionality reduction processing, then the map information is compiled into the potential space, and the obstacle information perception and feature extraction are carried out. (**b**) Based on the encoded map, the starting position, and target position, the optimal path is predicted. Among them, Pnet is a feedforward neural network containing multiple fully connected layers, ReLU activation layers, and dropout layers to prevent overfitting. (**c**) The intelligent sampling capability of MPNet is integrated into RRT* to perform bidirectional optimal path planning.

Figure 1 presents a comprehensive and iterative framework for enhancing path planning in three-dimensional urban environments, combining the strengths of RRT* for initial path generation and MPNet for optimization.

The following pseudo-code (see Algorithm 1) shows the core framework of the MPN-RRT* algorithm, including key links such as dimensionality reduction, mixed sampling, and path generation, so that the implementation process of the algorithm can be understood more intuitively.
**Algorithm 1** MPN-RRT* Framework**Require:** Start configuration $q_{start}$, Goal region *G*, Configuration space $C_{\mathrm{space}}$ **Ensure:** An optimized path *P* from $q_{start}$ to a configuration in *G*
  1:$Z\leftarrow \mathrm{DimensionalityReduction}(C_{\mathrm{space}})$         ▹ Environment Representation  2:$S_{MPN}\leftarrow \mathrm{InitializeMPNet}\left(Z\right)$              ▹ Initialize MPNet sampler  3:$T\leftarrow \mathrm{InitializeTree}(q_{start})$; $T.V\leftarrow \{q_{start}\}$; T.E←∅  4:**for** i=1 to *N* **do**  5:      **if** $\mathrm{rand}()<p_{mpn}$ **then**  6:            $q_{\mathrm{rand}}\leftarrow S_{MPN}\left(q_{start},q_{goal}\right)$        ▹ Use MPNet for informed sampling  7:      **else**  8:            $q_{\mathrm{rand}}\leftarrow \mathrm{UniformRandomSample}\left(C_{\mathrm{free}}\right)$      ▹ Fallback to uniform sampling  9:      **end if**10:      $q_{\mathrm{nearest}}\leftarrow \arg\min_{q\in T.V}∥q_{\mathrm{rand}}-q∥$11:      $q_{\mathrm{new}}\leftarrow \mathrm{Steer}\left(q_{\mathrm{nearest}},q_{\mathrm{rand}},\Delta q\right)$12:      **if** $\mathrm{IsCollisionFree}(q_{\mathrm{nearest}},q_{\mathrm{new}})$ **then**13:            $Q_{\mathrm{near}}\leftarrow \{q\in T.V|∥q-q_{\mathrm{new}}∥\leq r_{i}\}$, where $r_{i}=\min\left(\gamma {\left(\frac{\log i}{i}\right)}^{1/d},\eta \right)$14:            $q_{\mathrm{parent}}\leftarrow \mathrm{ChooseParent}\left(Q_{\mathrm{near}},q_{\mathrm{nearest}},q_{\mathrm{new}}\right)$15:            $T.V\leftarrow T.V\cup \{q_{\mathrm{new}}\}$; $T.E\leftarrow T.E\cup \{\left(q_{\mathrm{parent}},q_{\mathrm{new}}\right)\}$16:            $\mathrm{Rewire}(T,Q_{\mathrm{near}},q_{\mathrm{new}})$17:      **end if**18:**end for**19:$P\leftarrow \mathrm{ExtractPath}(T,q_{start},q_{goal})$20:**return** *P*


### 3.2. Perception Module

The first part of the framework is the perception module. At its core, it is an Enet with a contractive autoencoder (CAE), which is designed to compress the input map environments into a more compact form through an encoding scheme based on a basis point set approach [11]. This process yields a more streamlined representation of the environment compared to the original map. Given that real-world map environments are often large-scale and sparsely populated, encoding the environment prior to training helps to reduce the dimensionality of the training data. Consequently, this simplifies the computational demands and decreases the time required for effective network training.

The Contractive Autoencoder (CAE) is utilized to extract a latent-space representation, denoted as *Z*, from raw point cloud data $x_{\mathrm{obs}}\subset X_{\mathrm{obs}}$. The encoding and decoding processes within the CAE are represented by the functions $f(x_{\mathrm{obs}},\theta^{e})$ and $g(f\left(x_{\mathrm{obs}},\theta^{e}\right);\theta^{d})$, respectively, where $\theta^{e}$ and $\theta^{d}$ represent the parameters of the encoder and decoder approximation functions. The CAE is trained in an unsupervised manner, using an objective function that comprises two components: the reconstruction error, given by ${∥x-g\left(f\left(x\right)\right)∥}^{2}$, and a regularization term, $\lambda \sum_{ij}{\left(\theta_{ij}^{e}\right)}^{2}$, where λ is the regularization coefficient.

(1) $$L_{\mathrm{Encoder}}\left(\theta^{e},\theta^{d}\right)=\frac{1}{N_{\mathrm{obs}}}\sum_{x\in D_{\mathrm{obs}}}{∥x-g\left(f\left(x\right)\right)∥}^{2}+\lambda \sum_{ij}{\left(\theta_{ij}^{e}\right)}^{2}\;$$

Additionally, the dataset $D_{\mathrm{obs}}$ is composed of point cloud data points $x_{\mathrm{obs}}\in N_{\mathrm{obs}}$ collected from various workspaces. The incorporation of the regularization term strengthens the capacity of the feature space *Z*, defined as $f(x_{\mathrm{obs}})$, to adapt within the vicinity of the training samples. This process promotes the development of features that are both invariant and robust [2].

In terms of the model architecture, with particular emphasis on the encoding function—since the decoding function g(f(x)) serves as the inverse to $f(x_{\mathrm{obs}})$—the encoding unit is meticulously described. It consists of three fully connected linear hidden layers, each followed by an output linear layer. The outputs from these hidden layers are passed through a Parametric Rectified Linear Unit (PReLU) activation function [13], which enables a non-linear transformation of the data.

The core task of the perception module is to reduce the dimension of a complex 3D city map to a 2D map while retaining key obstacle information. This module simplifies the computational complexity through dimensionality reduction technology, and it provides an efficient input for the intelligent sampling module.

### 3.3. Intelligent Sampling Module

The second part of the framework is theintelligent sampling module. At its core, it is a Pnet with a deep sampler, structured as a feed-forward neural network. It consists of a feature input layer, multiple fully connected layers interspersed with dropout layers, and an output layer. The rectified linear unit (ReLU) function is utilized as the activation function for the fully connected layers. To evaluate the performance of the output layer, Pnet employs a weighted mean square distance metric as its loss function. This systematic architecture enables efficient processing and learning from the environmental data input to the network. The Pnet module processes the encoded environmental information, integrating both the starting and target positions, to predict a subsequent state that more closely approximates the desired endpoint. This network is trained using supervised learning, with optimal trajectories generated by conventional path planning methods serving as the training dataset.

#### 3.3.1. Deep Sampler

The deep sampler is a dynamic, forward-propagating deep neural network characterized by its stochastic nature and defined by a set of parameters θ. This advanced network is designed to process the encoded features of obstacles, denoted as *Z*, along with the robot’s state at a given time *t*, represented by $x_{t}$, and the target state $x_{\tau }$. Its core function is to generate a subsequent state, ${\hat{x}}_{t+1}$, which is strategically placed within the free navigable space $X_{\mathrm{free}}$. This state is optimized to guide an unmanned aerial vehicle (UAV) incrementally closer to its designated target area. The operational formula for this process is documented in Equation (2).

(2) $${\hat{x}}_{t+1}=\mathrm{DeepSampler}\left(\left(x_{t},x_{\tau },Z\right);\theta \right)$$

Within the training framework of DeepSMP, the RRT* algorithm is utilized to generate paths that closely approximate the optimal trajectory. These training trajectories are organized as sequences, or tuples, denoted by $\sigma^{*}=\left[x_{0},x_{1},\ldots ,x_{T}\right]$. This format ensures that each consecutive sequence of states within $\sigma^{*}$ represents a feasible and executable route. The primary objective of the training process is to minimize the mean squared error (MSE), thereby reducing the discrepancy between the predicted states ${\hat{x}}_{t+1}$ and the actual states $x_{t+1}$ as defined by RRT*. This is formalized in Equation (3):

(3) $$L_{\mathrm{MSE}}\left(\theta \right)=\frac{1}{N_{p}}\sum_{j}^{N}\sum_{i=0}^{T-1}{∥{\hat{x}}_{j,i+1}-x_{j,i+1}∥}^{2}\;$$

Here, $N_{p}\in N$ represents the total number of pathways $\tilde{N}$, multiplied by the individual lengths of these pathways.

Regarding the architecture of the model, the deep sampler features a multi-layered, twelve-layer neural network structure. Each internal layer is composed of a linear layer, combined with a PReLU mechanism [13,14] and a dropout feature [15,16], except for the final layer, which omits the dropout component. The twelfth and final layer serves as the output segment, taking inputs from the previous layer and transforming them to match the desired output size. This size is carefully adjusted to align with the dimensional requirements of UAV configurations. For instance, the dimensional specifications for various UAV models are as follows: a 2D point-mass UAV is set to 2, a 3D point-mass UAV to 3, a rigid-bodied UAV to 3, and a UAV with a 6 Degrees of Freedom (DOF) framework to 6. For all problem scenarios except the 6 DOF UAV trajectory planning, the input to the deep sampler consists of the obstacle representation *Z*, the UAV’s real-time state $x_{t}$, and the target state $x_{\tau }$. In the context of a 6-DOF environment, which is assumed to be singular, the input to the deep sampler simplifies to just the current state $x_{t}$ and the intended target state $x_{\tau }$.

During the training phase, the network adjusts its weights to minimize the weighted mean squared error between the predicted states and the actual states (referred to as ground truth). In the simulation and implementation phases, the initial state inputted into the system is considered the current state for the first cycle. In subsequent cycles, the most recently predicted subsequent state by the network becomes the new current state. This iterative process continues until the current state converges with the target goal state.

#### 3.3.2. Bidirectional Neural Planning Approach

At the same time, in this module, we use a bidirectional neural planning approach, which operates in both forward and backward directions. The Bidirectional Neural Planner (BNP) takes as input the spatial encoding of obstacles *Z*, the robot’s starting position $c_{\mathrm{init}}$, and its target destination $c_{\mathrm{goal}}$. It generates a bidirectional trajectory comprising a forward path $\sigma_{a}$ from the start to the goal and a backward path $\sigma_{b}$ from the goal to the start, with both paths incrementally approaching each other. Initially, $\sigma_{a}$ and $\sigma_{b}$ are initialized with the robot’s initial position $c_{\mathrm{init}}$ and the target position $c_{\mathrm{goal}}$, respectively. The development of these paths alternates sequentially, such that if $\sigma_{a}$ is extended during one iteration *i*, $\sigma_{b}$ will be extended in the subsequent iteration, with the roles of $\sigma_{a}$ and $\sigma_{b}$ switching after each iteration. Following each extension, an attempt is made to establish a direct connection between the two paths if possible.

To facilitate this connection, we utilize asteerTo function, which employs a greedy heuristic to join the paths. This approach is similar to the strategy used in RRT-Connect [17], where efforts are made to connect the two paths after each extension phase. If a successful connection between $\sigma_{a}$ and $\sigma_{b}$ can be established, the BNP constructs and outputs a unified trajectory θ, combining states from both $\sigma_{a}$ and $\sigma_{b}$.

During the sampling phase, at each time interval *t*, the system generates a routing task that includes the robot’s initial state $c_{\mathrm{init}}$, its target state $c_{\mathrm{goal}}$, and information on obstacles $\lambda_{\mathrm{obs}}$. Before deploying MPNet to plan a trajectory for a given problem, the network undergoes a training phase with expert demonstrations for up to $N_{c}$ iterations, where $N_{c}$ is a non-negative integer. If MPNet is unable to generate a feasible path or is not invoked, a proficient planner is used to identify a viable route σ. This expert-generated path σ is then stored in a replay buffer $B^{*}$ and a session-specific memory module *M*, both shaped by the chosen sampling strategy. MPNet refines its performance on the demonstrated scenario through a bounded optimization technique, as detailed in Equations [2,3]. Similar to lifelong learning practices, rehearsal sessions are conducted using archived samples.

Our planning framework (Pnet) is fundamentally probabilistic, incorporating dropout layers throughout its architecture. This inherent randomness is leveraged to produce a diverse set of informed samples, which serve as the basis for a traditional SMP’s sample generator. These samples are carefully selected to have a high likelihood of contributing to a viable and near-optimal trajectory, thereby enhancing the SMP’s ability to quickly and accurately identify solutions.

The following pseudo-code (see Algorithm 2) shows the key elements of the bi-directional search strategy for a more intuitive understanding of the implementation of the algorithm.
**Algorithm 2** Bidirectional Neural Planner**Require:** Start configuration $c_{start}$, Goal configuration $c_{goal}$, Environment representation *Z* **Ensure:** A feasible path σ
  1:$\sigma_{a}\leftarrow \left\{c_{start}\right\}$; $\sigma_{b}\leftarrow \left\{c_{goal}\right\}$           ▹ Initialize forward and backward paths  2:**for** i=0 to $N_{max}$ **do**  3:      $c_{\mathrm{pred}}\leftarrow \mathrm{Pnet}\left(Z,\mathrm{end}\left(\sigma_{a}\right),\mathrm{end}\left(\sigma_{b}\right)\right)$         ▹ Predict next configuration  4:      $\sigma_{a}^{\prime }\leftarrow \mathrm{Steer}\left(\mathrm{end}\left(\sigma_{a}\right),c_{\mathrm{pred}},\Delta_{c}\right)$  5:      **if** $\mathrm{IsFeasible}(\sigma_{a}^{\prime })$ **then**  6:            $\sigma_{a}\leftarrow \mathrm{Concatenate}\left(\sigma_{a},\sigma_{a}^{\prime }\right)$  7:      **end if**  8:      **if** $\mathrm{CanConnect}(\mathrm{end}\left(\sigma_{a}\right),\mathrm{end}\left(\sigma_{b}\right),\epsilon_{\mathrm{connect}})$ **then**  9:            $\sigma_{\mathrm{bridge}}\leftarrow \mathrm{LocalPlanner}\left(\mathrm{end}\left(\sigma_{a}\right),\mathrm{end}\left(\sigma_{b}\right)\right)$10:            **if** $\mathrm{IsFeasible}(\sigma_{\mathrm{bridge}})$ **then**11:                  $\sigma \leftarrow \mathrm{Concatenate}(\sigma_{a},\sigma_{\mathrm{bridge}},\mathrm{Reverse}\left(\sigma_{b}\right))$12:                  **return** PostProcess(σ)13:            **end if**14:      **end if**15:      $\mathrm{SWAP}(\sigma_{a},\sigma_{b})$               ▹ Swap roles for the next iteration16:**end for**17:**return** FAILURE


### 3.4. Path Generation Module

In order to further optimize the path planning process, this module proposes a hybrid sampling strategy, which combines MPNet intelligent sampling and uniform random sampling. Specifically, sampling points generated by MPNet are used with probability (p), and uniform random sampling is used with probability (1 − p):

(4) $$q_{\mathrm{sample}}=\left\{\begin{array}{cc} \mathrm{DeepSampler}(\left(x_{t},x_{\tau },Z\right);\theta ) & \mathrm{with}\;\mathrm{probability}\;p\\ \mathrm{UniformSample}() & \mathrm{with}\;\mathrm{probability}\;1-p \end{array}\right.$$

In addition, the RRT* algorithm ensures that the generated path is as close to the optimal solution as possible by connecting the newly generated node (q_ text new) to the nearest node (q_ text near) and optimizing the path cost. The optimized path cost calculation formula is as follows:

(5) $$C\left(q_{\mathrm{new}}\right)=\min_{q\in \mathrm{Near}(q_{\mathrm{new}})}\left(C\left(q\right)+\mathrm{Cos}t\left(q,q_{\mathrm{new}}\right)\right)$$

In order to improve the degree of the path, the path degree index (S) is introduced in this paper. Its calculation formula is as follows:

(6) $$S=\frac{1}{N-1}\sum_{i=1}^{N-1}∥\frac{v_{i+1}-v_{i}}{t_{i+1}-t_{i}}∥$$

Overall, the MPN-RRT* algorithm proposed in this paper significantly improves the efficiency of path planning through the combination of deep learning and sampling algorithms. The perception module simplifies the calculation complexity through dimensionality reduction processing, the intelligent sampling module generates more effective sampling points through deep learning, and the path generation module ensures the optimality and performance of the path through hybrid sampling strategies and path optimization. This performs well in complex 3D cities and provides an efficient solution for real-time autonomous navigation of UAVs.

### 3.5. Overall Process

**Figure 2:**
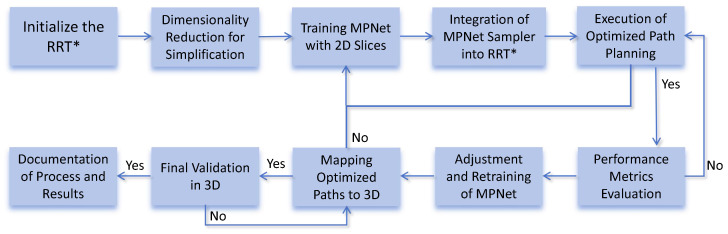
The execution of optimization.

Figure 2 presents the details of a sophisticated and iterative approach for enhancing path planning in three-dimensional urban environments, leveraging the synergy between RRT* for initial path planning and MPNet for optimization. This methodology is designed to incrementally refine path planning through a series of deliberate steps, each incorporating specific validation points to ensure the quality and efficacy of the optimization process. The detailed summary below captures each step’s essence and its role within the larger optimization workflow:

1. Initialization with RRT*: The process begins with RRT*, a path planning algorithm renowned for its ability to rapidly explore and identify feasible paths within complex spaces. This serves as the foundation for the initial path planning phase.
2. Dimensionality Reduction for Simplification: Given the inherent complexity of 3D maps, the map is decomposed into 2D planes to simplify the path planning process. The integrity of these 2D slices is rigorously verified to ensure they accurately represent the original 3D environment. If any integrity issues are detected, the process requires a return to the previous step for map adjustments or re-loading.
3. Training MPNet with 2D Slices: With the 2D slices prepared, MPNet is trained to navigate the urban environment efficiently. This training phase is critical for enabling MPNet to learn optimal pathfinding strategies. Successful training is essential for proceeding; otherwise, parameter adjustments and retraining are required.
4. Integrating MPNet Sampler with RRT*: Following successful training, MPNet’s sampling capabilities are integrated into RRT*, replacing its uniform sampling mechanism. This integration aims to enhance the RRT* algorithm by leveraging MPNet’s learned insights for more efficient pathfinding.
5. Optimized Path Planning Execution: With the MPNet sampler integrated, the system executes optimized path planning, carefully evaluating the stability of the algorithm and the feasibility of generated paths. Any issues with path feasibility trigger a return to the training phase (Step 3), emphasizing the iterative nature of the process for continuous improvement.
6. Performance Metrics Evaluation: The efficiency, length, and smoothness of the optimized paths are evaluated against predefined performance criteria. This step ensures that the optimization efforts meet the desired standards. If these criteria are not met, further optimization is required, prompting a return to the path planning execution phase (Step 5).
7. Adjustment and Retraining of MPNet: Depending on the outcomes of performance evaluations, adjustments may be necessary to enhance MPNet’s path planning capabilities. This could involve parameter tuning or algorithmic adjustments, followed by retraining. If improvements are not observed, the process iterates this step, emphasizing the commitment to achieving optimal results.
8. Mapping Optimized Paths to 3D: Successfully optimized 2D paths are then projected back onto the original 3D map to verify their correctness and applicability in the three-dimensional space. If this mapping is incorrect, the process necessitates a return to the dimensionality reduction phase (Step 2) for correction.
9. Final Validation in 3D Environment: The ultimate step before finalization is the validation of optimized paths within the 3D environment, ensuring that the optimization process has been successful. Failure in this validation leads to a return to the adjustment and retraining phase (Step 7), underscoring the iterative, quality-driven nature of the entire process.
10. Documentation of Process and Results: This final step involves the comprehensive documentation of the entire process, including each phase, findings, adjustments, and conclusions. This documentation serves not only as a record of the optimization efforts but also as a guide for future optimization endeavors.

This detailed flowchart illustrates the methodology employed in this study, showcasing a systematic and iterative optimization process that integrates deep learning technology with established path search algorithms to achieve superior path planning in complex urban environments. This approach is characterized by its emphasis on continuous improvement, validation, and adaptability at every step to ensure that the final path planning solution meets the highest standards of efficiency and feasibility.

### 3.6. 3D Environment Setup (Simple and Complex)

For the development of our three-dimensional maps, two unique virtual environments were designed, with dimensions of 200 × 200 × 200 and 800 × 800 × 200, respectively. These maps emulate urban scenarios tailored for unmanned aerial vehicle (UAV) motion planning tasks. The larger map features a higher density of obstacles to enhance the complexity and realism of the environment.

#### Map Construction Approach

In constructing the maps, a grid-based methodology was employed, dividing the space into discrete cells classified as either navigable or obstructed. The arrangement and dispersion of these obstacles were carefully crafted to reflect the quintessential characteristics of urban environments, including buildings, streets, and natural barriers. This design aims to provide a multifaceted and realistic experimental framework.

The smaller map features a relatively sparse distribution of obstacles, representing an expansive urban setting with broad pathways and minimal detours required. In contrast, the larger map contains a denser arrangement of obstructions, including complex clusters of buildings and narrow passages. This configuration mimics the intricate navigation challenges found in densely populated urban areas, such as downtown districts or historic neighborhoods. It is designed to test and validate the adaptability and efficiency of motion planning algorithms in environments with varying obstacle densities.

Additionally, the larger map incorporates several no-fly zones within its airspace, strategically placed to increase navigational complexity for UAVs. These zones simulate real-world scenarios where drone operations may be restricted due to safety, privacy, or security concerns. The inclusion of no-fly zones adds another layer of complexity to the simulations, enhancing the realism of the test environment and ensuring that the algorithms can navigate not only around physical obstacles but also comply with regulatory constraints.

To further enhance the realism of the simulations, the obstacles within the maps vary not only in their horizontal distribution but also in their vertical elevation, reflecting the diverse heights of urban structures. This requires motion planning algorithms to perform sophisticated three-dimensional navigation, avoiding obstacles both horizontally and vertically.

**Figure 3:**
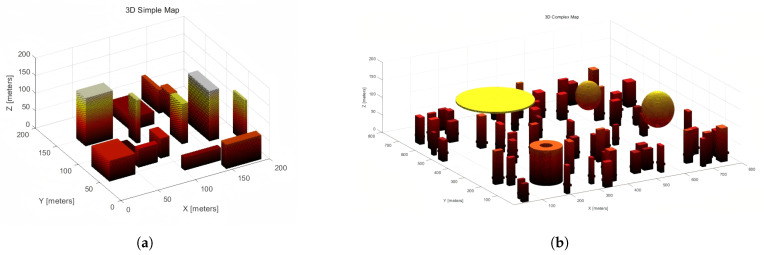
(**a**) 200 × 200 × 200 3D simple map. (**b**) 800 × 800 × 200 3D complex map.

The 200 × 200 × 200 and 800 × 800 × 200 3D urban maps are depicted in Figure 3 below.

## 4. Simulation and Results

This section thoroughly examines the real-world application and efficacy of the proposed MPNet model via meticulously designed experiments. It delineates the experimental setup, model training procedures, operational details, and a comparative evaluation of the results. The objective is to elucidate the model’s performance, applicability, and limitations. By meticulously analyzing the outcomes, this segment endeavors to validate theoretical assertions and highlight the model’s practical viability and potential drawbacks.

### 4.1. Experiment Setup

To systematically assess the efficacy of the proposed MPNet deep learning model in enhancing the uniform sampler of RRT*, we performed extensive simulation evaluations and analyses. In the context of UAV path planning, there is currently no universally accepted standard environmental model. For sampling-based path planning approaches, a fundamental configuration is typically employed to depict obstacles or threats within the scene. Consequently, in this study, we utilized rectangular and cylindrical models to approximate real-world urban structures and obstacles, while spherical and hemispherical models were employed to represent warning radars, threat zones, or no-fly areas. However, reliance on basic configurations inevitably leads to a significant loss of terrain information, particularly when converting a 3D map into a two-dimensional maze map through slicing and reduction. To mitigate this issue, an elevation map was generated in this study to more accurately reflect terrain-related threat information.

Table 1 and Table 2 illustrate some key parameter values for the basic MPNet and RRT* models, where Case 1, Case 2, and Case 3 correspond to different weight distributions within the flight cost function. Additionally, the parameters for radars, anti-aircraft guns, and towers are also indicated.

The orientation quaternion of a UAV’s initial and terminal states denotes the UAV’s initial orientation within a three-dimensional space. A quaternion is a four-dimensional vector capable of encoding any rotation in 3D space. It is widely favored in 3D programming and aerospace applications over alternative representations such as Euler angles, as it circumvents the issue of gimbal lock and offers a smooth, continuous description of orientation.

A quaternion comprises one real component and three imaginary components, commonly denoted as $q=(q_{w},q_{x},q_{y},q_{z})$ or q=(w+xi+yj+zk).

In UAV applications, the real component *w* corresponds to the cosine of half the rotation angle. The imaginary components *x*, *y*, and *z* represent the sine of half the rotation angle scaled by the respective components of the rotation axis.

The orientation quaternions of a UAV’s starting and ending states define the vehicle’s attitude (pitch, roll, and yaw) in a 3D environment at the onset or conclusion of its maneuver. These quaternions must be normalized to ensure unit length, thereby representing a valid rotation. The UAV’s control system relies on this information to maintain and adjust its orientation during flight, facilitating precise navigation towards its intended destination.

### 4.2. Model Training

In the subsequent section, we delve into the methodology of converting three-dimensional slices into maze maps, a technique that effectively simplifies intricate spatial data into a more tractable format. Furthermore, we will address the enhancement of the pre-existing model, referred to as “mpnSE2”. Our focus here will be on the detailed optimization of the model’s functionality and the assurance that its performance is closely aligned with our navigation goals across diverse and complex environments.

#### 4.2.1. Dataset

This section offers a detailed examination of the methodologies employed in generating the datasets essential for model training. The initial dataset comprises 3D maps and path generation information for 100 and 300 paths, which have not undergone smoothing. The starting and target point coordinates for these paths were randomly selected within a predefined range around the experimental starting and target points. This approach may potentially limit the model’s generalization capabilities.

To prepare for training our motion planning model, we slice the comprehensive 3D urban maps into a series of horizontal sections, each representing a unique 2D plane at varying elevations. This strategic slicing, from z=1 to z=200, divided into 21 discrete sections, serves a dual purpose. The rationale behind this slicing process stems from the need to render the problem more computationally tractable. By decomposing the voluminous data into manageable segments, we can intricately capture the complexities of urban architecture, such as multi-story buildings and varied terrains, within manageable layers. This allows for focused analysis and training on specific aspects of urban navigation, one altitude stratum at a time.

The methodology adheres to a systematic and meticulous slicing protocol: The 3D map is first subdivided along the Z-axis into 21 equidistant horizontal planes. Each plane is then abstracted into a 2D representation, where the nuances of elevation are temporarily set aside to prioritize lateral navigational elements. These 2D planes are further simplified into maze maps, preserving navigational complexity while removing the third dimension. This step enhances the algorithm’s ability to learn essential pathfinding skills within a 2D context, ensuring fundamental navigational strategies are solidified before tackling the full 3D environment.

The advantages of this approach are as follows:

- **Enhanced Focus**: Training within a 2D maze map context enables the algorithm to focus on essential pathfinding strategies without the initial burden of vertical considerations.
- **Efficiency**: It accelerates the training process by reducing the computational overhead associated with processing 3D data.
- **Layered Learning**: The approach facilitates a layered learning experience, where lessons learned in one stratum can inform decisions in subsequent layers.

**Figure 4:**
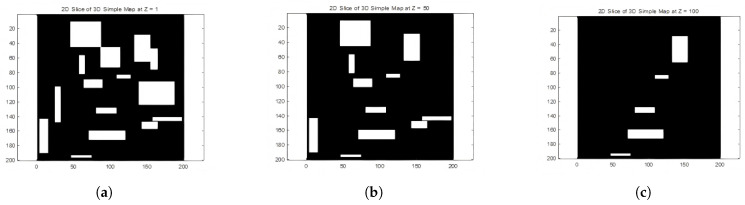
Slices of 3D simple map.

However, reducing 3D maps to a 2D maze format may result in the loss of important vertical (Z-axis) information, both in terms of landscape features and path elevation changes. When remapping these paths back to 3D space, the Z-axis data needs to be manually reintegrated to accurately reconstruct the vertical progression of the paths. Figure 4a–c illustrate a 3D simplified city map of the sliced sections (z=1, z=50, z=100).

When a three-dimensional urban map is sliced at a specific height and the slice contains no buildings, the resulting two-dimensional image predominantly displays the ground or empty spaces. In such cases (e.g., z=150, z=200), the visual outcome of the slicing process typically manifests as a grayscale image. Each pixel value within this grayscale image corresponds to height information or other attributes of the sliced surface, such as reflectivity. However, due to the absence of three-dimensional structures like buildings, the image may appear relatively uniform and featureless.

**Figure 5:**
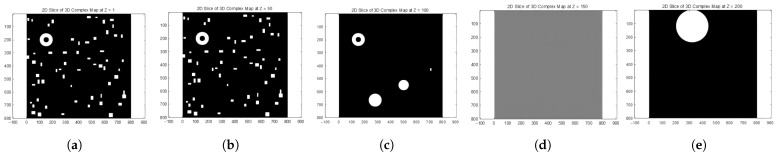
Slices of 3D complex map.

Similarly, the slices of a 3D complex map are shown below (Figure 5a–e).

Following the slicing procedure, the slices corresponding to each Z height layer in the 3D city map are transformed into maze maps. This transformation is a crucial step in preparing the dataset for training the sampler-learning model.

#### 4.2.2. Training

Table 3 presents the interface of the original pre-trained deep learning network analyzer, which provides a detailed overview of the architecture and parameter settings of a particular neural network. The network comprises 12 layers, primarily consisting of an input layer, fully connected layers, ReLU activation layers, and dropout layers.

1. Input Layer (input): Receives input data with 108 features.
2. Fully Connected Layers (fc1–fc4): Four fully connected layers with 256, 128, 64, and 32 neurons, respectively, each with corresponding weights and biases.
3. ReLU Activation Layers (relu1–relu4): Four activation layers using ReLU (Rectified Linear Unit) as the activation function.
4. Dropout Layers (dropout1, dropout2): Used to prevent overfitting by randomly shutting down some neuron activations during training.
5. Output Layer (output): A fully connected layer with four neurons. the output size is 4(C) × 1(B), which likely represents four categories for a classification problem.

The “Learnable Sizes” column displays the size of the weights and biases for each fully connected layer, which are parameters the model needs to learn during training. The “State Sizes” column does not display any information, as the state of these layers does not change at runtime. Only the training results of the complex map model are shown below. The learning rate is equal to 0.001.

**Figure 6:**
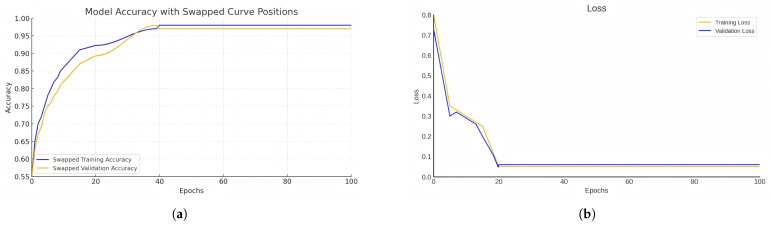
(**a**) Model accuracy. (**b**) Model loss.

As depicted in Figure 6, the model’s training accuracy and validation accuracy both increase with the number of epochs. In the initial stages of training, the accuracy rises rapidly, reflecting the model’s capacity for swift learning. After approximately 20 epochs, both accuracies achieve high stability, with the final training accuracy nearing 100%. Notably, the validation accuracy closely tracks the training accuracy, suggesting that the model exhibits strong generalization capabilities on unseen data.

Figure 6 also illustrates the changes in loss value as training progresses. The loss value quantifies the discrepancy between the model’s predictions and the actual labels. A decreasing loss value indicates improved prediction accuracy. The figure shows that both training and validation losses decline sharply in the early epochs and then smoothly approach zero, further validating the effectiveness of the model training.

We also evaluated the performance of the model using unseen data, where the perceptual part of MPNet has strong ability to cross different generalizations. Many algorithms need to be reconfigured or recalibrated in each new one, and MPNet-SMP, a pre-training module in the MPNet architecture, can be applied to unknown ones once trained, showing strong generalization capabilities. At the same time, the MPNet-guided RRT* model architecture also shows short-term performance in the path, which can be used in real-time planning of paths in the real world.

In summary, the model demonstrates robust learning and generalization capabilities throughout the training process, with no evident signs of overfitting or underfitting. These two figures collectively confirm the stability and high accuracy of the proposed model, laying a solid foundation for subsequent research. In subsequent experimental verification, the trained MPN-RRT* can effectively generate and efficiently fly paths, and it shows good robustness and adaptability in two different settings. At the same time, our experimental tests on simple and complex datasets also show that MPN-RRT* performs better than others, which further supports the effectiveness and feasibility of the MPN-RRT* algorithm.

### 4.3. Results

This section will conduct path planning for RRT*, MPNet, and MPN-RRT* in both simple and complex maps that have been generated, and it will analyze various metrics including path length, program execution time, path smoothness, and UAV flight duration. Additionally, it will explore the factors contributing to the differences among various sampling-based algorithms.

Following the completion of the final path generation, to enhance the UAV’s path planning process, it is necessary to smooth the generated path to address certain limitations of the basic RRT* algorithm. The paths generated by RRT* are often characterized by sharp edges and corners, which are not suitable for UAV navigation. This highlights the need for path smoothing, which involves optimizing the trajectory by transforming the waypoints into a smoother curve. This process aims to minimize the rate of change of jerk, or ’snap,’ which is essential for ensuring fluid UAV motion. By focusing on minimizing snap, we can ensure that the UAV’s movements are continuous and free from abrupt changes in acceleration.

To quantify the smoothness of a path defined by 3D coordinate points in robotics and control applications, the following steps can be taken:

- **Compute Velocity**: Determine the velocity vectors by calculating the differences between adjacent coordinate points along the path.
- **Compute Acceleration**: Obtain acceleration vectors by finding the differences between consecutive velocity vectors.
- **Assess Smoothness**: Use the average magnitude of the acceleration vectors, typically measured using the Euclidean norm, as a metric for smoothness. A path with a lower average acceleration magnitude is deemed smoother.

This approach provides a fundamental method for evaluating path smoothness, focusing on the changes in acceleration to assess the variability along the path.

#### 4.3.1. Simple Map

**Figure 7:**
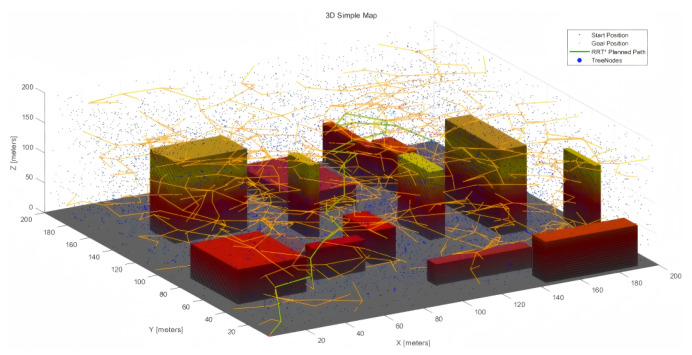
RRT* planned path for the simple map.

**Figure 8:**
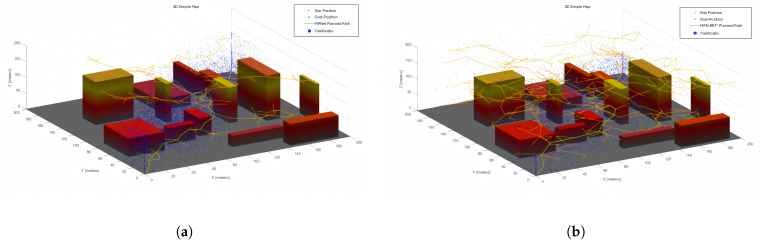
(**a**) MPNet planned path for the simple map. (**b**) MPN-RRT* planned path for the simple map.

Figure 7 and Figure 8 show the RRT*, MPNet, and MPN-RRT* planned path for the simple map below.

**Figure 9:**
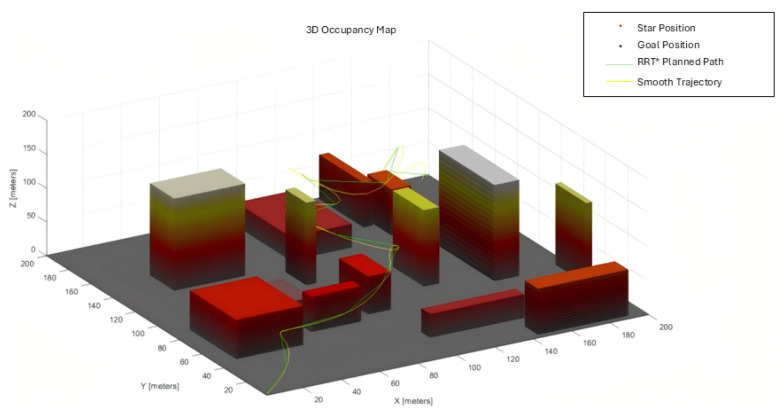
Smoothness of RRT* planned path.

**Figure 10:**
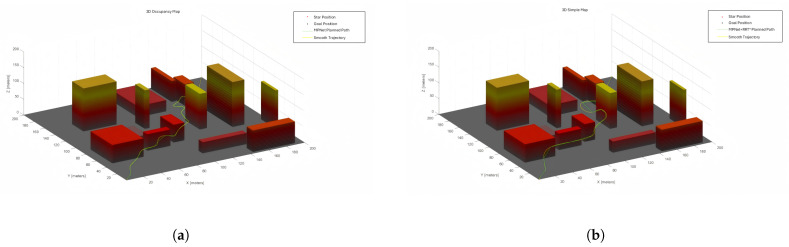
(**a**) Smoothness of MPNet planned paths. (**b**) Smoothness of MPN-RRT* planned path.

Utilizing the aforementioned method for calculating smoothness, we have evaluated the smoothness of the generated path. Figure 9 and Figure 10 illustrate the original generated path and the smoothed path, respectively. For ease of comparison, the visualization of TreeNodes and tree expansion has been omitted.

Table 4 below shows the data parameter information of each algorithm in a simple map environment.

#### 4.3.2. Complex Map

**Figure 11:**
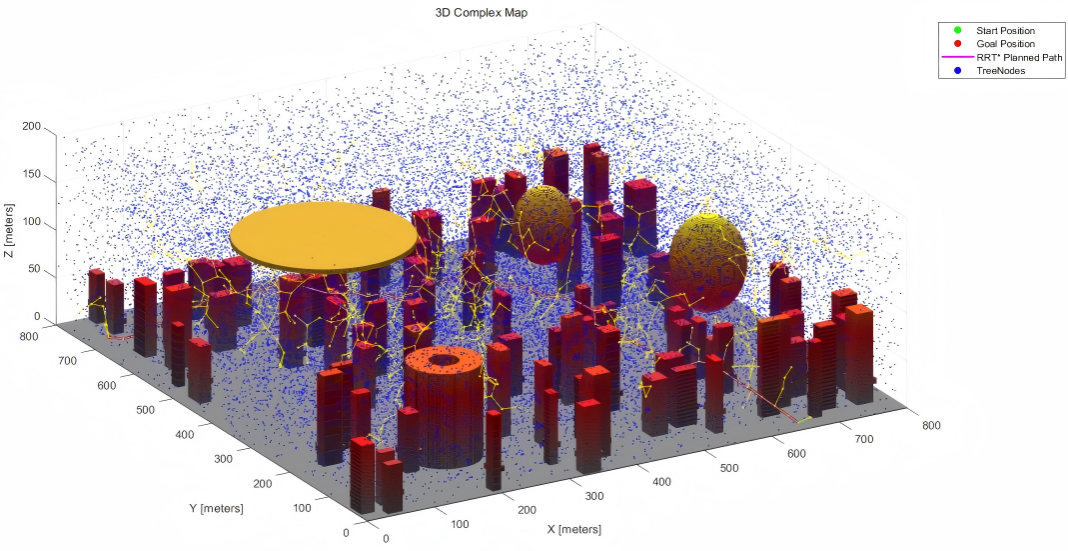
RRT* planned path for the complex map.

**Figure 12:**
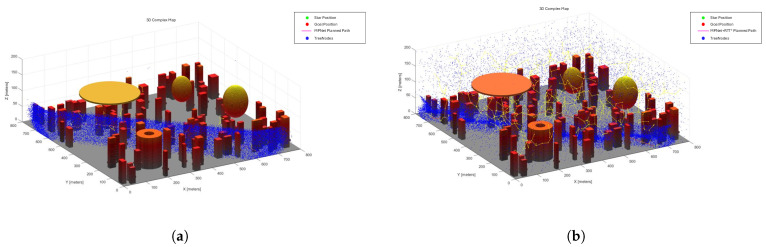
(**a**) MPNet planned path for the complex map. (**b**) MPN-RRT* planned path for the complex map.

Figure 11 and Figure 12 show the RRT*, MPNet, and MPN-RRT* planned path on the simple map below.

**Figure 13:**
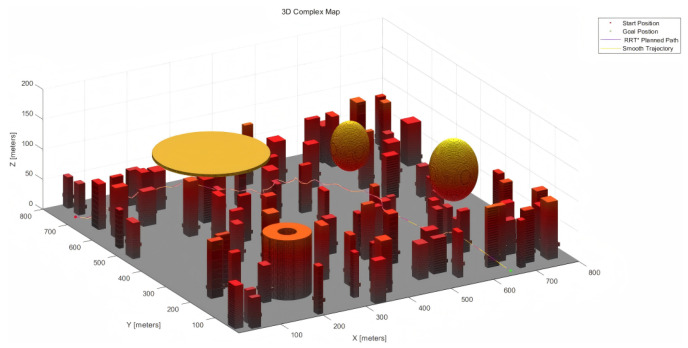
Smooth RRT* planned path.

**Figure 14:**
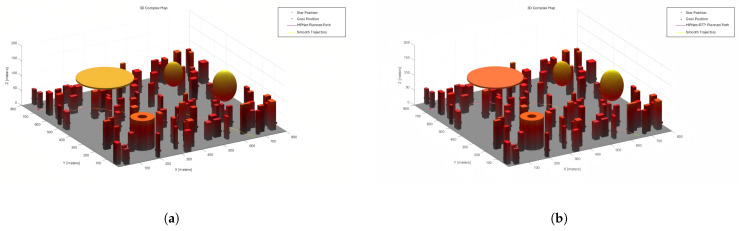
(**a**) Smoothness of MPNet planned path. (**b**) Smoothness of MPN-RRT* planned path.

Employing the previously described method, we assessed the smoothness of the generated path. Figure 13 and Figure 14 depict the original path and the smoothed path, respectively. To enhance the clarity of comparison, the visualization of TreeNodes and tree expansion has been omitted.

Table 5 below provides the data parameter information of each algorithm in a complex map environment.

### 4.4. Analysis

As illustrated in the preceding section, the six simulation figures depict the path planning performance of the three algorithms across two distinct maps. These figures provide immediate visual insights into various elements, including the planned path, sampled points (TreeNodes), partial tree expansion, start and end points, and the smoothed path. In terms of quantitative data, for the simple map, Table 4 reveals that MPNet exhibits a notable edge in the simulation experiments. Its planning time is significantly reduced to 46.77 s, which is approximately 47.8% less than the 89.58 s required by RRT*. The planning time for MPN-RRT* also decreases to 75.30 s, representing a reduction of about 15.9% compared to RRT*. Regarding path length, MPNet achieves a planned path of 381.76 m, which is roughly 19.8% shorter than RRT*’s 476.23 m. The path length for MPN-RRT* is 393.62 m, about 17.3% shorter than RRT*. Flight time data further corroborate these findings, with MPNet completing the flight path in only 127.25 s; approximately 19.8% faster than RRT*’s 158.74 s. MPN-RRT* requires 131.21 s, which is about 17.3% faster than RRT*. In summary, in the simple map simulation, the MPNet algorithm outperforms the other two algorithms in both efficiency and path optimization. MPN-RRT* occupies a middle ground, while RRT* demonstrates relatively weaker performance. These results suggest that MPNet may be a more suitable choice for applications demanding rapid and efficient path planning. The optimization outcomes of MPN-RRT* also reflect notable improvements across various metrics compared to RRT*.

For the complex map, Table 5, which details the planning simulations on the complex map, shows that MPNet significantly outperforms the other algorithms in terms of efficiency and path optimization. The data indicate that MPNet achieves a planning time of 124.0951 s, which is considerably faster than both RRT*’s 151.9875 s and MPN-RRT*’s 142.3021 s, highlighting a more efficient planning process. In terms of path length, MPNet’s planned path of 1009.9985 m is substantially shorter than RRT*’s 1174.9542 m, demonstrating superior route optimization. The smoothness of the path, which reflects the degree of deviation, is optimal for MPNet at 5.6000, indicating a more refined trajectory compared to other methods. The flight time, a practical measure of efficiency, shows that MPNet completes the course in 336.6662 s, faster than both RRT* and MPN-RRT*, emphasizing its overall superiority in speed and directness. These findings suggest that MPNet is the most effective algorithm for scenarios requiring rapid and efficient route creation, with MPN-RRT* offering a balanced middle ground and RRT* lagging behind in performance.

## 5. Discussion

### 5.1. Interpretation of Results

The findings presented in Section 4 offer a compelling demonstration of MPNet’s exceptional performance in 3D urban map planning simulations. The reduced planning time achieved by MPNet holds dual significance: it underscores the algorithm’s capacity to rapidly compute flight paths, a critical feature for time-sensitive applications, and it also implies enhanced computational efficiency, which may translate into energy savings in practical implementations. The shortened path length not only reduces the distance traveled but also decreases flight time, a crucial factor for battery-powered UAVs where energy conservation is paramount.

MPNet’s high smoothness score serves as a robust indicator of its ability to generate more direct and potentially safer flight paths, avoiding unnecessary maneuvers that could strain UAV components or increase risk in obstacle-rich environments. This aspect highlights the algorithm’s capability to create more reliable and predictable trajectories, which is especially advantageous in congested or complex airspace scenarios.

The decreased flight time is not merely a measure of speed but also a reflection of overall operational efficiency. This improvement could have substantial implications for sectors that rely on UAVs for delivery services, surveillance, or search-and-rescue missions, where time is a critical factor. Faster flight times can lead to increased throughput and service delivery, potentially revolutionizing the deployment of aerial services.

Moreover, MPNet’s consistent performance in these simulations suggests that it is well-adapted to dynamic environments requiring real-time decision-making, positioning it as a preferred solution for advanced UAV applications. The improvements demonstrated by MPN-RRT* over RRT* alone also indicate that hybrid approaches can achieve meaningful advancements in path planning, offering a balanced integration of the strengths of different algorithms.

In summary, these findings underscore the importance of algorithm selection in UAV path planning and highlight the tangible benefits of choosing an algorithm that excels in both efficiency and path optimization. As UAV usage continues to expand across various industries, the insights gained from these results may drive future research and development efforts, leading to even more optimized and intelligent path planning solutions.

### 5.2. Strengths and Limitations

#### 5.2.1. Strengths

MPNet demonstrates substantial advantages in terms of path planning speed and efficiency, as evidenced by its performance in complex map simulations. The algorithm achieves a notable reduction in planning time, indicating that it is both swift and capable of rapidly converging to a solution. Additionally, MPNet excels at optimizing path length, resulting in shorter and more direct routes that correspond to decreased UAV flight times. Importantly, this efficiency does not compromise path quality, as MPNet generates the smoothest paths among the tested algorithms, suggesting less erratic or convoluted routing.

MPN-RRT* offers a hybrid approach that leverages the strengths of both MPNet and RRT*. By combining the rapid convergence of MPNet with the comprehensive exploration capabilities of RRT*, it achieves improved planning times and path lengths compared to RRT* alone. This makes it a potentially suitable choice for applications that require a balance between planning speed and thoroughness.

RRT* is a well-established algorithm known for its robustness and effectiveness in navigating highly complex spaces. Widely used across various applications, it is valued for its thorough exploration capabilities and ability to find paths in challenging environments where other algorithms may struggle.

#### 5.2.2. Limitations

While MPNet demonstrates superior performance, it may face limitations in scenarios where computational resources are constrained. Its efficiency might stem from more intricate calculations, which could pose challenges in environments with limited processing power. Additionally, MPNet’s reliance on neural networks implies a need for substantial training data, which may not always be readily available or practical to obtain. The hybrid MPN-RRT* algorithm, despite enhancing RRT*’s performance, may still retain some of RRT*’s computational inefficiencies. The integration of the two methods could also introduce greater complexity in terms of parameter adjustment and algorithm setup, potentially complicating its implementation for some users or applications.

Of course, we also acknowledge the current limitations of the model, particularly in terms of generalization across diversity and dynamics. Future mission directions will also strive to enhance this adaptability, refine the robustness of the model, and tailor its learning process to the nuances of specific scenarios. This may involve fostering a more diverse training dataset while incorporating real-time feedback and experimenting with further incremental learning strategies.

## 6. Conclusions and Future Work

### 6.1. Summary of Findings

The incorporation of MPNet into the RRT* framework has yielded significant improvements in UAV path planning for complex urban environments. This novel integration has notably enhanced path efficiency, markedly reduced pathfinding time, and produced smoother trajectories. As a result, it has considerably strengthened UAVs’ navigational capabilities in intricate terrains.

In the context of complex path planning, our research underscores the critical role of deep learning. MPNet’s predictive capabilities effectively augment RRT*’s sampling strategies, creating an intelligent and adaptive pathfinding approach. The fusion of traditional pathfinding techniques with advanced neural network models has paved the way for more intelligent and reliable UAV missions.

It is important to address the limitations observed in our experiments, particularly regarding the model’s generalization capabilities. These constraints, primarily attributed to the dataset’s size and reliance on pre-trained models, highlight areas for future work. To improve generalization, future research could focus on expanding the dataset and developing a bespoke 3D model from the ground up. This direction is essential for the model to effectively manage a broader range of scenarios with enhanced accuracy and reliability.

### 6.2. Future Work

As we look to the future, numerous research opportunities present themselves. One particularly noteworthy direction is to examine how MPNet can be adapted to various other pathfinding algorithms, assessing its influence on efficiency and reliability across different contexts. The potential applications of MPNet are not limited to urban environments; it could also transform navigation in rural, industrial, and even extraterrestrial landscapes.

We recognize the current limitations of the model, especially in terms of its ability to generalize across a wide range of diverse and dynamic environments. Enhancing the MPN-RRT* framework to handle dynamics will be a key direction of our research in future studies. We envisage an effective strategy to integrate real-time sensor data to dynamically update maps and obstacle locations. This will allow the MPN-RRT* algorithm to respond to new information and path plans accordingly. For example, by integrating data from LiDAR or cameras, systems can detect moving obstacles in real time and update their positions in maps. The MPN-RRT* algorithm can then re-plan the path to avoid these dynamic obstacles, ensuring safe and efficient navigation. In addition, predictive models can be used to predict the movement of dynamic obstacles, enabling algorithms to proactively plan paths to avoid potential future collisions. We expect that this combination of real-time data integration and predictive modeling will effectively improve the adaptability of the framework to dynamic scenarios, making it more suitable for obstacles and changing real-world UAV path planning.

Through continued research and development, we anticipate a future in which drones can achieve unprecedented levels of autonomous navigation, leading to safer airspace and more efficient services. Progress in this domain is expected to have a significant impact, advancing the frontiers of autonomous navigation and robotics.

## Figures and Tables

**Table 1:** Basic parameter values.

**Parameter**	**Validation Distance**	**Max Iterations**	**Max Connection Distance**	**Goal Bias**	**View**	**Urban Obstacle Width**	**Urban Obstacle Length**	**Urban Obstacle Height**
Value	0.01	2500	0.35	0.2	(−10,55)	(1,50)	(1,50)	(1,150)
**Parameter**	**Start Orientation Quaternion**	**Goal Orientation Quaternion**	**TreeNodes (simple)**	**TreeNodes (complex)**	**Simple_ start**	**Simple_ goal**	**Complex_ start**	**Complex_ goal**
Value	(0.7,0.2,0,0.1)	(0.3,0,0.1,0.6)	10,000	30,000	(1,1,2)	(199,199,2)	(21,700,10)	(650,20,2)

**Table 2:** Basic parameter values.

	**Center**	**StartHeight**	**EndHeight**	**InnerRadius**	**OuterRadius**
**Auti-air-1**	(120,320,Z)	195	200	1	120
	**Center**	**StartHeight**	**EndHeight**	**Radius**	**Radius**
**Auti-air-2**	(550,500,100)	60	140	40	40
	(666,280,100)	50	150	50	50

**Table 3:** Pre-trained deep learning network analyzer.

**Name**	**input**	**fc1**	**relu1**	**dropout1**	**fc2**	**relu2**
**Type**	Feature Input	Fully Connected	ReLU	algs.mpnetDrop	Fully Connected	ReLU
**Activations**	108(C)*1(B)	256(C)*1(B)	256(C)*1(B)	256(C)*1(B)	128(C)*1(B)	128(C)*1(B)
**Name**	**dropout2**	**fc3**	**relu3**	**fc4**	**relu4**	**output**
**Type**	algs.mpnetDrop	Fully Connected	ReLU	Fully Connected	ReLU	Fully Connected
**Activations**	128(C)*1(B)	64(C)*1(B)	64(C)*1(B)	32(C)*1(B)	32(C)*1(B)	4(C)*1(B)

**Table 4:** Parameters of simple map planning.

	UAV Speed	Planning Time	Path Length	Smoothness of Path	Smoothness of Smooth Path	TreeNodes in pthObj	Flying Time
RRT*	3 m/s	89.578327 s	476.2304 m	14.6655 m/s^2^	1.38831 m/s^2^	427	158.743467 s
MPN-RRT*	3 m/s	75.302104 s	393.6166 m	21.4137 m/s^2^	0.95496 m/s^2^	1023	131.205533 s
MPNet	3 m/s	46.773859 s	381.7558 m	10.1742 m/s^2^	1.29150 m/s^2^	1518	127.251933 s

**Table 5:** Parameters of complex map planning.

	UAV Speed	Planning Time	Path Length	Smoothness of Path	Smoothness of Smooth Path	TreeNodes in pthObj	Flying Time
RRT*	3 m/s	151.987594 s	1174.9542 m	13.3808 m/s^2^	7.2766 m/s^2^	1004	391.6514 s
MPN-RRT*	3 m/s	142.302104 s	1064.6041 m	11.9616 m/s^2^	3.9062 m/s^2^	1928	354.8680 s
MPNet	3 m/s	124.095057 s	1009.9985 m	5.6000 m/s^2^	4.0076 m/s^2^	2862	336.6662 s

## Data Availability

Data are contained within the article.
